# Marked Microevolution of a Unique *Mycobacterium tuberculosis* Strain in 17 Years of Ongoing Transmission in a High Risk Population

**DOI:** 10.1371/journal.pone.0112928

**Published:** 2014-11-18

**Authors:** Carolina Mehaffy, Jennifer L. Guthrie, David C. Alexander, Rebecca Stuart, Elizabeth Rea, Frances B. Jamieson

**Affiliations:** 1 Public Health Ontario, Toronto, Canada; 2 University of Toronto, Toronto, Canada; 3 Saskatchewan Disease Control Laboratory, Regina, Canada; 4 Toronto Public Health, Toronto, Canada; St. Petersburg Pasteur Institute, Russian Federation

## Abstract

The transmission and persistence of *Mycobacterium tuberculosis* within high risk populations is a threat to tuberculosis (TB) control. In the current study, we used whole genome sequencing (WGS) to decipher the transmission dynamics and microevolution of *M. tuberculosis* ON-A, an endemic strain responsible for an ongoing outbreak of TB in an urban homeless/under-housed population. Sixty-one *M. tuberculosis* isolates representing 57 TB cases from 1997 to 2013 were subjected to WGS. Sequencing data was integrated with available epidemiological information and analyzed to determine how the *M. tuberculosis* ON-A strain has evolved during almost two decades of active transmission. WGS offers higher discriminatory power than traditional genotyping techniques, dividing the *M. tuberculosis* ON-A strain into 6 sub-clusters, each defined by unique single nucleotide polymorphism profiles. One sub-cluster, designated ON-A^NM^ (Natural Mutant; 26 isolates from 24 cases) was also defined by a large, 15 kb genomic deletion. WGS analysis reveals the existence of multiple transmission chains within the same population/setting. Our results help validate the utility of WGS as a powerful tool for identifying genomic changes and adaptation of *M. tuberculosis*.

## Introduction

Globally, tuberculosis (TB) is an important cause of morbidity and mortality. World-wide, *M. tuberculosis* is responsible for more than 1 million deaths per year. In low incidence countries, homeless/under-housed individuals represent one of the groups at greater risk for TB infection and disease [1]–[5]. TB outbreaks within homeless settings have been documented throughout North America [6]–[9].

One endemic strain, designated Ontario A (ON-A), has been circulating since at least 1997 in the urban homeless/under-housed population of Toronto, Canada and has been responsible for TB outbreaks in 2001 and 2004 with new cases identified every year [5], [10]. Genotyping is an essential component of epidemiological investigations. However, the *M. tuberculosis* ON-A isolates are defined by a unique combined spoligotype and 24-locus MIRU-VNTR (24-MIRU) profile, while IS*6110* RFLP generates pseudo-clusters among these strains [10].

Although several reports have highlighted the epidemiological value of whole genome sequencing (WGS) over traditional genotyping techniques [11]–[17], presently only a few studies have evaluated the utility of WGS to study whole genome changes of *M. tuberculosis* during long-term continuous active transmission [15].

We evaluated the usefulness of WGS to retrospectively validate and identify transmission events associated with TB cases due to *M. tuberculosis* ON-A over the last 17 years. We used a phylogenetic analysis based on single nucleotide polymorphisms (SNP) to portray the microevolution of this *M. tuberculosis* strain during almost two decades of on-going transmission in a high risk population. Our analysis revealed the presence of six independent transmission chains and the presence of an ON-A natural mutant, defined by a large genomic deletion that most likely emerged during the first ON-A TB outbreak in 2001.

## Methods

### *M. tuberculosis* clinical isolates

*M. tuberculosis* isolates were obtained from clinical specimens routinely received at the Public Health Ontario Laboratories for TB diagnosis. All available isolates from 1997–2013 with genotypes consistent with the ON-A strain [6], [18] were selected. All isolates were susceptible to all first line drugs.

The work described in this manuscript relates directly to improvement of routine TB surveillance and outbreak management, therefore research ethics board (REB) approval was not required.

### DNA extraction

Genomic DNA (gDNA) was extracted as previously described [19] with minor modifications [20].

### Genotyping

24-locus MIRU-VNTR [21], spoligotyping [22] and IS*6110* RFLP [23] were performed using standard methods, and data were analyzed with BioNumerics v6.1 (Applied Maths, St-Martin Latem, Belgium). RFLP patterns were compared as previously described [10].

### Whole genome sequencing

DNA was prepared for sequencing as described elsewhere [20]. Illumina paired-end reads were trimmed using quality scores and then aligned to *M. tuberculosis* H37Rv reference genome (NC_000962.2) using the CLC Genomics workbench (v.6.0.2) software. For 5 of the 61 isolates, quality and/or quantity of the DNA were not suitable for WGS and therefore these were not included in the analysis.

Accuracy of WGS assembly and analysis workflow was assessed by sequencing the H37Rv reference strain that is used in our clinical lab (Material S1).

### Variant calling

Single nucleotide polymorphisms (SNPs) and small insertion-deletion (indel) events were identified using a probabilistic variant detection with cutoffs of a minimum read depth of 20X and a variant frequency of at least 75. Indels were not considered for any further analyses. SNPs were further filtered by removing positions associated with PE, PPE and PE_PGRS gene families which have been previously shown to represent false positives and due to their high variation are not suited for phylogenetic analysis [17]. SNPs unique to any of the fifty-six high quality whole genome sequences were manually inspected in each individual alignment for accuracy and all ambiguous results were discarded.

### Phylogenetic analysis

**Figure 1:**
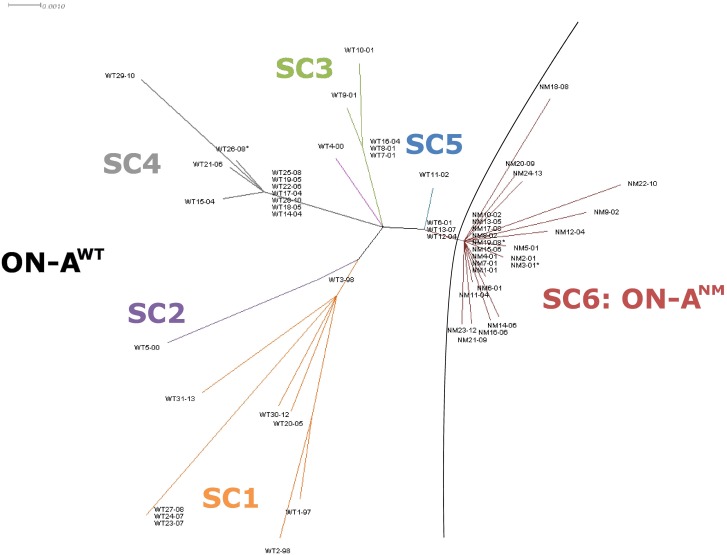
ON-A Phylogenetic tree constructed in Splitstree ver. 4. All 722 polymorphic sites were included. Phylogenetic tree was built using BioNJ algorithm and then filtered for parsimony-informative sites.

A concatemer of the SNPs was generated and then used to reconstruct the phylogeny of ON-A using SplitsTree v.4 software [24] and the BioNJ algorithm [25]. Trees were then re-constructed using the Equal Angle algorithm [26] with equal-daylight and box opening optimization [27] available in SplitsTree v.4 (Figure 1).

### Demographic and clinical data

Demographic characteristics and clinical information for each TB case was obtained from Ontario’s integrated Public Health Information System (iPHIS) as well as responsible Public Health Units (Table 1). This information is routinely recorded by Public Health Units for all laboratory and clinically confirmed TB cases. Data was anonymized and all personal information removed from the final data set. Time lapse between onset of symptoms and diagnosis as well as treatment start date were not available for most cases and therefore were not included in this study.

**Table 1:** Demographic characteristics of ON-A TB cases.

Variable		All Individuals (n = 57)	ON-A^WT^ Individuals (n = 33)	ON-A^NM^ Individuals (n = 24)
Age (years)				
	Mean age (± std dev)	50.0 (±12.9)	51.4 (±11.6)	48.2 (±14.4)
	Range	20.3–74.3	23.8–74.3	20.3–72.5
Gender				
	Female	5	3	2
	Male	52	30	22
Birthplace				
	Born in Canada	40	22	18
	Born outside Canada	11	5	6
	Unknown	6	6	0
Disease Classification				
	Pulmonary	49	27	22
	Extra-pulmonary	2	1	1
	Pulmonary with extra-pulmonary involvement	6	5	1
Mortality (TB as cause of deathψ)				
	Yes	3	3	0
	No	10	5	5
	Unknown	6	5	1
Housing Status				
	Under-housed	43	23	20
	Housed	4	2	2
	Unknown	10	8	2
Risk Factors				
	HIV/AIDS	8	4	4
	Injectable drug use	10	4	6
	Alcohol abuse	31	17	14

ψ Number of deaths during or shortly after completing TB treatment.

### Social network visualization

Known epidemiological/social connections identified during routine public health contact investigation (i.e roommates, close friends, used same drop-in, etc.) among forty-seven ON-A cases were available in iPHIS. The igraph [28] package of R (v3.0.2) was used to generate the analysis. Transmission events were defined based on the genomic and epidemiological information (i.e. SNP pattern, contact information, year of diagnosis and infectiousness based on smear and chest x-ray results).

## Results

In this study we performed the whole genome sequencing of 61 *M. tuberculosis* isolates identified during routine genotyping as members of a large cluster denominated ON-A and spanning 17 years (1998–2013). The 61 isolates corresponded to 57 patients, most of whom were homeless/under-housed individuals (75.5%). The epidemiological and molecular typing characteristics of this cluster up to 2008 have been published elsewhere [5], [6]. Table 1 summarizes the demographic characteristics of all 57 patients.

Isolates in this cluster are characterized by a highly similar 24MIRU and spoligotype patterns while RFLP analysis of the ON-A strain results into three pseudo-clusters defined by RFLP types A, B and C. The main 24MIRU pattern has not been reported in the international MIRU database (http://www.miru-vntrplus.org/MIRU/index.faces) and personal communication with the corresponding health authorities of the other Canadian provinces indicates that this genotype is unique to Toronto’s inner city population.

**Figure 2:**
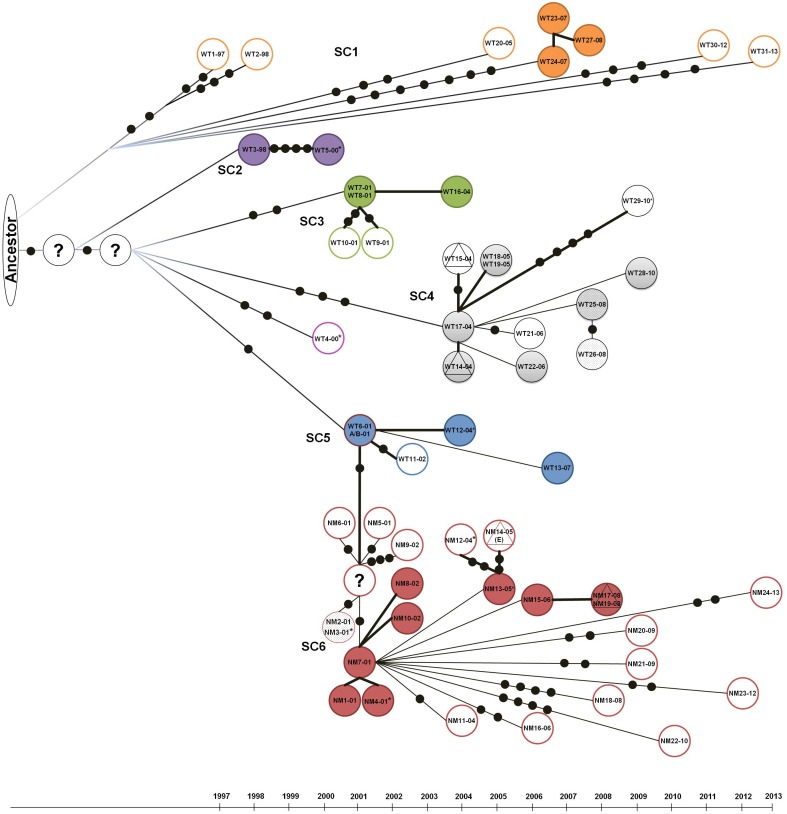
Transmission events of ON-A TB cases based on WGS information and epidemiologic analysis. Colored solid circles represent TB isolates that are identical to the suspected primary case (0 SNPs). Open circles represent related cases, separated by black solid dots representing SNPs acquired over time. Triangles represent TB cases outside of the homeless/under-housed group. Isolates from the same TB patient are represented by circles with dots in their background: WT25-08 and WT26-08; NM2-01 and NM3-01; and NM17-08 and NM19-08. *Isolates that had more than one passage prior to WGS.

Of the 61 *M. tuberculosis* isolates, WGS was successfully performed in 56 isolates, corresponding to 53 TB cases and further sub-divided the large ON-A group into 6 different sub-clusters (SC1-6) (Figure 1 and 2).

**Figure 3:**
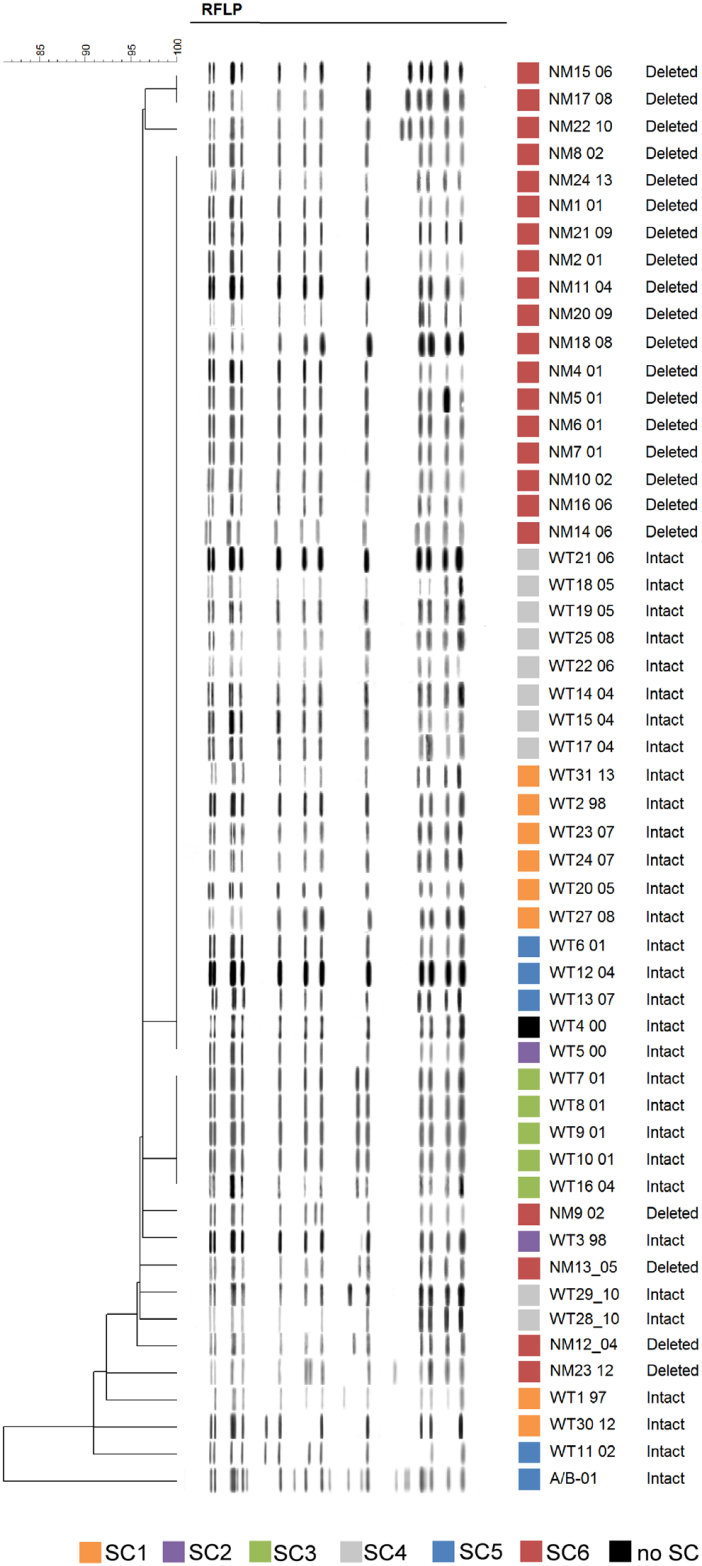
Dendrogram of IS*6110* RFLP showing the 15 kb deletion distribution. Branches indicate the clusters with identical RFLP patterns. Colored squares represent each of the six sub-clusters identified by WGS. Information regarding the presence or absence of the 15 kb deletion for each isolate is shown as “deleted” or “intact”.

Figure 3 combines the RFLP and SNP-clustering results for all 53 cases with final WGS data. Most isolates belong to RFLP type A (n = 34) followed by RFLP B (n = 5) and RFLP C (n = 3). Nine isolates were not clustered by RFLP and 1 presented a mixed pattern corresponding to an infection with strain ON-A and strain ON-B, which is also commonly found in the homeless/under-housed population. Figure 3 illustrates the lack of correlation between RFLP and SNP-clustering with the exception of RFLP type B which corresponded to SC4.

Sub-cluster classification was based on SNP analysis using as reference, the genome of laboratory strain H37Rv. In summary, 722 SNPs were identified in ON-A isolates when compared with H37Rv. Of these, 641 SNPs, including 333 (52%) non-synonymous, 224 (35%) synonymous, and 84 (13%) non-coding SNPs, were conserved in all sequenced isolates and only served to differentiate H37Rv from the ON-A strain. The remaining 81 SNPs were variable among the sequenced isolates and were used to identify sub-cluster associations.

Most isolates belonged to the most recently emerged sub-cluster SC6 (n = 24), followed by SC4 (n = 10), SC1 (n = 8), SC5 (n = 5) and SC2 (n = 2). One isolate was not placed in any of the 6 sub-clusters.

Of the 81 SNPs, 2 were present in the majority of ON-A isolates, except for most isolates in SC1. One SNP was only present in SC-6 and one was present in both SC4 and SC5 isolates. The remaining 77 SNPs were either sub-cluster associated (i.e. present in two or more isolates) (17/77) or singletons (60/77).

### Sub-clusters, SNPs and transmission events

Phylogenetic analysis identified 6 ON-A sub-clusters (Figure 1). One isolate did not group with any of the identified sub-clusters. Phylogenetic analysis and temporal association of isolates from the ON-A strain suggests the independent emergence of SC1–SC5 prior to 1997 and subsequent clonal expansion of sub-clusters SC3, SC4 and SC5 (Figures 1 and 2). Contrary to this, SC6 appears to have emerged in 2001 during the first TB outbreak associated with ON-A.

SNP patterns of isolates belonging to SC1 group suggest they represent infections acquired from a common ancestor(s) prior to 1997 and most likely represent re-activation of latent TB infection rather than immediate secondary cases. The exceptions were two isolates, (WT24-07 and WT27-08) with SNP patterns identical to the source isolate WT23-07. The two individuals, from whom the isolates WT23-07 and WT24-07 were obtained, lived in the same rooming house, and both used the same drop-in location which was associated with the two ON-A TB outbreaks. Interestingly, the individual associated with isolate WT27-08 was diagnosed in a different jurisdiction north of Toronto, but is clearly related to the other two isolates and demonstrates the high mobility of this population.

Contrary to SC1, all other sub-clusters had a clear source case. In group SC2, isolate WT5-00 gained 4 SNPs in approximately 2 years after infection from WT3-98. In group SC3, the source case (WT07-01) resulted in two other primary TB cases in the same year and a later reactivation case in 2004.

In SC4, the source case (WT17-04) resulted in secondary cases in shelter workers (both in 2004). Five additional cases with an identical SNP pattern as the suspected source case were also identified in subsequent years (2005–2010). In addition, the source was also genetically related to a case in 2006 with 1 SNP gained and another case in 2010 with 4 additional SNPs. It is not possible to determine if the case in 2010 (WT29-10) originated from the source case or from one of the five cases with identical SNP pattern to the source. However, the large number of SNPs suggests that WT29-10 corresponds to a re-activation from an infection acquired in 2004 or 2005. This is also supported by epidemiological data that indicates WT29-10 may have shared a common workplace with WT17-04 at the time this source case was ill.

24-MIRU, spoligotype and RFLP indicated that A/B-01 represented a mixed infection with two strains commonly found in this population (ON-A and ON-B) [6] and therefore its WGS data was not included in the phylogenetic analysis. However, manual inspection of all 81 variable SNP positions in the genome alignment generated for this isolate demonstrated the presence of all SC5 SNPs, allowing us to include the A/B-01 case within the SC5 group.

The two suspected source cases in group SC5 (WT6-01 and A/B-01) resulted in two subsequent cases in 2004 and 2007, both of which have identical SNP patterns to the suspected source(s). In addition, these two cases were the most likely source for the emergence of SC6 in 2001.

It is possible that the high bacteria burden present in WT6_01 or the A/B-01 mixed case (smear 2+ and 3+ respectively) contributed to the emergence of SC6. Both patients also had abnormal chest x-rays and together with their smear results suggest they were highly infectious. The chest x-ray of the mixed A/B case also showed cavitation. Social network analysis demonstrated the large number of contacts shared by these two patients, including individuals in both ON-A^WT^ and ON-A^NM^ groups (Figure S1).

The SC6 group was very homogeneous. SNPs within this group were mostly singletons and represented temporal acquisition of substitutions in single isolates. Nine SC6 isolates (37.5%) had no accumulation of SNPs in an 8 year time frame (Figure 2).

Because of the high number of isolates with identical SNP patterns in SC6, it was not possible to determine the exact transmission chain in this group. Multiple control measures to reduce spread of TB in Toronto’s homeless shelters were implemented as a consequence of the outbreaks in 2001 and 2004. This provides support to the idea that most cases after 2005 correspond to reactivation of infections acquired during one of the two TB outbreaks. The only exception is case NM17-08 which is an individual outside of the high risk group for which contact investigations indicate the source case for that individual was NM15-06. For the remaining cases, the most likely index case was NM7-01 who was highly infectious, with smear +3 and abnormal chest x-ray.

### WGS revealed a large genomic deletion in SC6

We discovered a large genomic deletion of more than 15 kb, comprising 12 genes in 26/61 (43.5%) ON-A isolates (Table 2), dividing the ON-A strain into two groups, ON-A^WT^ and ON-A^NM^ representing presence or absence of the 15 kb genomic region, respectively (Figure 1, Table 2). SC1–SC5 belong to the ON-A^WT^ group while isolates in SC6 are all ON-A^NM^. In contrast to ON-A^NM^, the ON-A^WT^ variant was very heterogeneous, characterized by the presence of multiple sub-clusters. In addition, thirty-two ON-A^WT^ SNPs were singletons while 16 were sub-cluster associated. ON-A^WT^ isolates also presented 3 additional SNPs within the deletion region. These SNPs were not included in the phylogenetic analysis. The genomic deletion was confirmed by a custom PCR in all isolates, including 5 for which WGS data was not available (Material S1). Sanger sequencing of the ON-A^NM^ PCR amplicons demonstrated that the insertion sequence IS*6110* is present and flanked by an incomplete Rv1358 at the 5′-end and by an incomplete Rv1371 at the 3′-end. Sanger sequencing of the flanking areas of the 15 kb deletion region in ON-A^WT^ also confirmed the presence of IS*6110* at the 3′ end of the region (upstream of Rv1371).

**Table 2:** Genes present in the 15 kb deleted region.

Deletedgene	Function	Annotations	Reference
Rv1358	Probable transcriptionalregulatory protein	LuxR family signature, expressionregulated by **SigF**; deleted in someclinical isolates	[33], [49]
Rv1359	Probable signaltransductionregulatory protein	Adenylate cyclase, family 3(Sensory pathways)	
Rv1360	Probable oxidoreductase	Expressed during GP infections;mRNA down-regulated in starvation	[50], [51]
Rv1361	PPE19 - Unknown	Expressed during GP infections;mRNA down-regulated instarvation; regulated by PhoP	[50]–[52]
Rv1362c	Unknown	Expressed during GP infections;membrane protein	[50]
Rv1363c	Unknown	None	
Rv1364c	Possible sigma factor Fregulatory protein	May be a capreomycin target;responds to heat stress	[53]
Rv1365	Anti-anti sigma factor F	Regulated by Redox potential	
Rv1366	Unknown	Homology to RelA-SpoTsuperfamily (ppGpp synthesis)	
Rv1367c	Possibly involved in cellwall metabolism	Homology to PBPs, B-lactamases	
Rv1368	Lipoprotein	None	
Rv1371	Unknown	Probably conserved membrane protein	

Heterogeneity resulting from IS*6110*-mediated deletion events during active TB infection has previously been reported in an individual with a very high bacteria burden [29] and it is possible that the high bacteria burden present in the two possible source cases (WT6-01 and A/B-01) as described previously could have contributed to the emergence of the deletion and origin of the ON-A^NM^ variant.

## Discussion

### Microevolution and adaptation events of *M. tuberculosis* during active TB transmission

We analyzed a cluster of *M. tuberculosis* isolates (ON-A) associated with on-going TB transmission in a large urban setting and its inner-city homeless/under-housed population. In order to evaluate the evolution of this *M. tuberculosis* strain during a 17 year period, we performed WGS analysis for all available ON-A isolates. We discovered a large genomic deletion (>15 kb) in a subset of ON-A isolates that divided the ON-A strain into two strain variants (ON-A^WT^ and ON-A^NM^). This large genomic deletion, which is present in 43% of the ON-A isolates demonstrates the presence of two distinct groups independent of the RFLP pattern.

Our phylogenetic and epidemiological analyses suggest this deletion emerged in 2000 during the first TB outbreak caused by ON-A.

The 15 kb deletion comprises a cluster of 12 genes, of which at least 5 code for regulatory proteins. Regulatory proteins govern the expression of clusters of genes involved in specific molecular pathways and therefore are ultimately responsible for the ability of the cell to adapt and survive in new environments. Two of the deleted genes identified in the ON-A^NM^ correspond to regulators of the Sigma-factor F (SigF). Sigma factors bind to RNA polymerase and alter its promoter preference resulting in subsets of genes that are differentially expressed upon environmental stresses to which a particular sigma factor responds. *M. tuberculosis* SigF is induced by nutrient starvation [30] and it is involved in the late stages of the disease [31]. One of the anti-sigF deleted genes (Rv1364c) strongly interacts with sigF and also with the sigF antagonist RsbW [32]. Other regulatory genes identified in the 15 kb deletion are the LuxR family regulator (Rv1358) which is also regulated by SigF [33], a signal transduction regulatory gene (Rv1359) and a conserved gene (Rv1366) in which we identified a domain representative of RelA-SpoT superfamily, responsible for the regulation of ppGpp concentration. ppGpp is a modified nucleotide used by bacteria as intracellular messengers that respond to different environmental stresses [34], [35]. In *M. tuberculosis*, ppGpp responses are associated with virulence and long-term survival [35], [36].

The presence of the insertion sequence IS*6110* at the site of the deletion suggests this event was IS*6110* mediated. Reports elsewhere have shown that IS*6110* mediated deletion events are common in *M. tuberculosis* [29], [37], [38] and even though the identification of a large genomic deletion in this highly related *M. tuberculosis* strain is very interesting, it should not be surprising given the high IS*6110* mediated mutation rate (transposition/generation) [39]. It has been proposed that although deleterious IS*6110* mediated mutations may occur frequently, these mutants are rapidly purged from the population by purifying selection [39]. Contrary to this, the 15 kb deletion seems to have emerged during the first TB outbreak in 2001; and since then the ON-A^NM^ has established itself among the homeless/under-housed population in Toronto.

Other examples of non-deleterious deletions exist. An *M. tuberculosis* isolate from the CAS family bearing a chromosomal deletion, was responsible for a large TB outbreak in Leicester, UK and was shown to have a lower inflammatory phenotype and higher intracellular growth similar to the hypervirulent strain HN878 [40]. An IS*6110*-mediated deletion was also reported elsewhere in an individual with disseminated TB and a high bacterial burden [29]. The deleted isogenic strain in this individual was only present in the lymph nodes and although transmission events were not identified, it is clear that the deletion did not impair the bacilli to replicate and cause disease.

Although the physiological effect of the ON-A^NM^ 15 kb deletion is presently unknown, based on the information available for the deleted genes, five of which have some role in gene regulation under environmental stresses; this deletion may have a direct impact on several molecular pathways and could enhance the capacity of this variant to spread and cause disease.

Conversely, the 15 kb deletion observed in ON-A^NM^ is in a hotspot position for IS*6110* transposition events [41], [42] and the genes located in this region may not be essential for the bacteria to transmit and cause disease. This would support the theory that the ON-A^NM^ was randomly fixed in the homeless/under-housed population, possibly supported by higher transmission rates in congregate setting such as shelters and a high prevalence of co-morbidities). It is possible that a weakened immune system allows for the appearance of potentially “unfit” mutations or large deletion events such as those reported here.

Studies focusing on fitness assessment and mining of the proteome and transcriptome of ON-A variants to determine the role of the deleted genes in the physiology and virulence of *M. tuberculosis* are currently underway.

### WGS as tool to determine TB transmission dynamics

Although RFLP, MIRU-VNTR and spoligotype are all powerful genotyping techniques, they cannot establish chronology of infection and lack resolution in long-term transmission events [43]–[46]. In contrast WGS has the potential for resolving the transmission dynamics of TB infection and when complemented with epidemiological data, it is an excellent tool to identify individual transmission events, and its use is very valuable in highly mobile communities such as the homeless/under-housed.

In addition to providing relevant and detailed information that can be used to construct and/or validate the transmission dynamics of a given strain, WGS also provides important information regarding SNPs, indels and larger genomic structural variants that can provide insights into the physiological characteristics of *M. tuberculosis* as a whole as well as particular characteristics of the strain of interest.

Recent genomics studies have highlighted the benefits of whole genome sequencing over traditional genotyping to uncover the transmission dynamics and molecular-guided TB control and surveillance [11], [12], [14]–[17]. For instance, Gardy et al. [12] demonstrated the usefulness of overlapping WGS data and social network analyses during outbreak investigations. The authors were able to determine transmission dynamics of 32 *M. tuberculosis* outbreak isolates in a period of two and half years. Similar to our findings, their SNP analysis revealed two lineages suggesting not one, but two simultaneous chains of transmission. The SNP calling algorithm in Gardy’s study did not include filtering out PE/PPE genes. This resulted in a higher genetic diversity than expected, but it is remarkable the similar findings with our study vis-à-vis the identification of multiple strain variants and clusters with otherwise identical RFLP and MIRU-VNTR patterns in a high risk population. Unlike the study by Gardy et al. [12] in which the two strain variants diverged and circulated in the community well before the outbreak, the ON-A^WT^ variant appears to have evolved during the 2001 TB outbreak and the emergent ON-A^NM^ was rapidly fixed in Toronto’s homeless/under-housed population.

Most WGS studies related to TB transmission dynamics have been focused on TB outbreaks in short time frames (1–30 months) [11]–[14], [16]. In all cases WGS represented an invaluable tool to either support or complement directionality of transmission based on epidemiological contact studies. A few long-term studies have also been conducted. Roetzer *et al*. [17] performed a prospective study to evaluate the correlation of WGS analyses with contact tracing data. Eighty-six *M. tuberculosis* isolates from a 13 year time frame were sequenced. WGS analysis confirmed the clonal expansion of an outbreak strain that was initially seen in a specific social setting in Hamburg, Germany, but slowly spreading outside of this particular setting [17]. In contrast to our findings, SNPs were the primary source of genome evolution during transmission and all small deletions and insertions detected were constant in all isolates.

Walker et al. [15] also confirmed the high power and resolution provided by WGS and determined the genomic diversity between patients in MIRU-VNTR community clusters in a period spanning 1–12 years.

Here, we studied the dynamics of TB disease over 17 years of on-going transmission in a homeless/under-housed population. Previous studies have demonstrated that large deletions and hundreds of polymorphic sites can exist in isolates with identical IS*6110* and MIRU-VNTR patterns [15]. As expected, we confirmed that clusters obtained by IS*6110* RFLP typing were not in agreement with the WGS-based division of ON-A isolates into two major groups based on the 15 kb large genomic deletion).

In addition, we identified 81 SNPs that are variable within the ON-A strain and further subdivide the strain into 6 distinct sub-clusters.

The large heterogeneity observed in our study group resulted in an average of 1.5 SNPs/case (81 SNPs/55 *M. tuberculosis* isolates) is slightly larger than those reported in the majority of *M. tuberculosis*-WGS studies which ranged from 0–1.3. However, in those studies, the larger variation rates were associated to community clusters (as opposed to household groups) spanning more than 10 yr of transmission [13], [15], [17]. Only two studies have shown larger variability. Cluster 9 in the study reported in Walker et al. (2013) [15] which was associated with substance abuse and presented an average of 2 SNPs/case in a 9 yr span; and the large cluster in Gardy et al. (2011) with a rate of more than 5 SNPs/case likely due to the inclusion of hypervariable PPE/PE genes [12].

Our rate of 1.5 SNPs/case may be a result from the long time span as well as the likely possibility of on-going transmission prior to 1997 and thus potential for missed links/cases. Although information regarding delay in diagnosis and adherence to treatment was not available, these are conditions likely to be encountered in a high risk setting such as the homeless/under-housed and could increase the possibility of bacilli evolution. For instance, SNP patterns of isolates belonging to SC1 group suggest they all represent infections acquired from a common ancestor prior to 1997 and most likely represent re-activation of latent TB infection with independently gained substitutions during latency ranging from 3–7 SNPs per event.

This hypothesis is supported by a study of TB latency in macaques that suggests *M. tuberculosis* mutation rate is fairly similar in active and latent TB, with the rate in latent TB being slightly higher [47]. In contrast, a recent study of latency in humans suggests a lower mutation rate during latency when compared to active TB [48]. However, this study only included two subjects with a long period (>10 yr) of latent infection, and as the authors suggested, it is possible that a broader spectrum of mutation rates exists during latent TB.

Certainly, our study supports this idea, as we were able to show both high heterogeneity as observed in SC1, but also several cases with a low SNP fixation (0–2 SNPs/yr) in the other ON-A groups particularly SC4–SC6 in which latency periods of more than 5 years and zero SNP changes were observed. For instance, in the ON-A^NM^ (SC6), some of the isolates obtained in 2008 did not present any additional SNPs when compared to isolates obtained in 2001. Similarly, group SC4 of ON-A^WT^ was represented by identical isolates from 2004 to 2010 and SC5 included isolates with identical SNP pattern from 2001 to 2007.

Although most SC1 cases are probably reactivations, we strongly believe that the high heterogeneity is not only due to latency but also to missing links within the group. This could be due to unrecognized/undiagnosed TB cases, TB cases ultimately diagnosed outside the province of Ontario, or to TB cases diagnosed prior to 1997 for which no isolates are available. Gaps in the PHOL genotyping database are another factor. Our laboratory implemented IS*6110* RFLP in 1997 but, until the introduction of a semi-automated MIRU-VNTR and spoligotyping program in 2007, Ontario did not have universal genotyping and only a subset of strains were analyzed or archived. Currently, the PHOL database includes patterns for >4000 isolates, but data for hundreds of strains obtained between 1997 and 2007 were never obtained.

In summary, the large number of singletons observed in our study, and the absence of progenitors for some SCs, are most likely due to gaps in our strain collection and genotyping database. However, SNPs present in some isolates may represent substitutions that originated during latency. In group SC6, the large majority of cases resulted from the presence of a super-spreader (NM7-01), and this suggestion is supported by clinical findings (e.g. smear 3+ and abnormal chest x-rays) consistent with a highly infectious state. Although it is possible that the high bacterial burden in this case could have contributed to the emergence and spread of isolates with differences of 2–4 SNPs, the large heterogeneity observed in these isolates is also compatible with a more plausible explanation such as mutations originating after secondary infection and long latency periods.

## Conclusion

We performed a large retrospective and longitudinal study which resulted in the characterization of the microevolution of a unique *M. tuberculosis* strain associated with a high-risk group population. Despite a clustered genotype pattern based on the combination of 24-MIRU and spoligotype, we identified a large genomic deletion in nearly half of the sequenced isolates and were able to identify the emergence of this deletion event to have occurred during the first TB outbreak caused by ON-A in 2001. Even though loss of large genomic regions is a major source of variation in *M. tuberculosis* [49], to the best of our knowledge, this is the first study in which identification of such a region was pinpointed during active TB transmission. Furthermore, we identified a larger than expected heterogeneity resulting from the microevolution of ON-A in 17 years of transmission and further delineated this strain into 6 distinct sub-clusters.

WGS has been proposed as a “gold standard” for strain typing in *M. tuberculosis* [12], [13], [15]. Our study confirms the value of WGS to determine transmission dynamics and isolate relatedness in a large cluster of on-going TB transmission extending over many years in a high risk population. Nonetheless, the large number of shared contacts between TB cases, the mobility of homeless/under-housed individuals, and TB latency contributed to the complexity of determining individual events of TB transmission in this population.

The peculiarities of our study cohort are noticeable: high risk cases, on-going transmission despite control measures, high mobility of cases and likely missing links. All of these factors could be associated with a higher than average microevolution dynamic resulting in high variability and multiple transmission chains. Our intention is not to make general conclusions adapted to other transmission environments but to decipher the high clonal complexity and microevolution rates that could be expected in a transmission event in a complex population.

## Supporting Information

Figure S1 **Social network analysis of ON-A subjects.** Social network analysis was performed using R statistical software (v3.0.2) with the igraph package. Each large circle represents a single individual colored by their sub-cluster as determined by WGS SNP analysis. Grey lines represent common contacts between study individuals and thick black lines represent direct epidemiological/social-connections between study individuals.
(PDF)

Material S1 **Supplementary methods and results section.** The supplementary methods describe the PCR amplification of regions flanking the 15 kb deletion. The supplementary results describe the WGS results of our laboratory strain H37Rv and how these results were use to evaluate the accuracy of our SNP calling algorithm. Table S1 in Material S1 shows the primers used for PCR amplification of regions flanking the 15 kb deletion.
(DOCX)
