# Supplementary figures and images for: Marked Microevolution of a Unique Mycobacterium tuberculosis Strain in 17 Years of Ongoing Transmission in a High Risk Population
Source: PLoS One. 2014 Nov 18;9(11):e112928. doi: 10.1371/journal.pone.0112928 (PMC4236100; doi:10.1371/journal.pone.0112928)

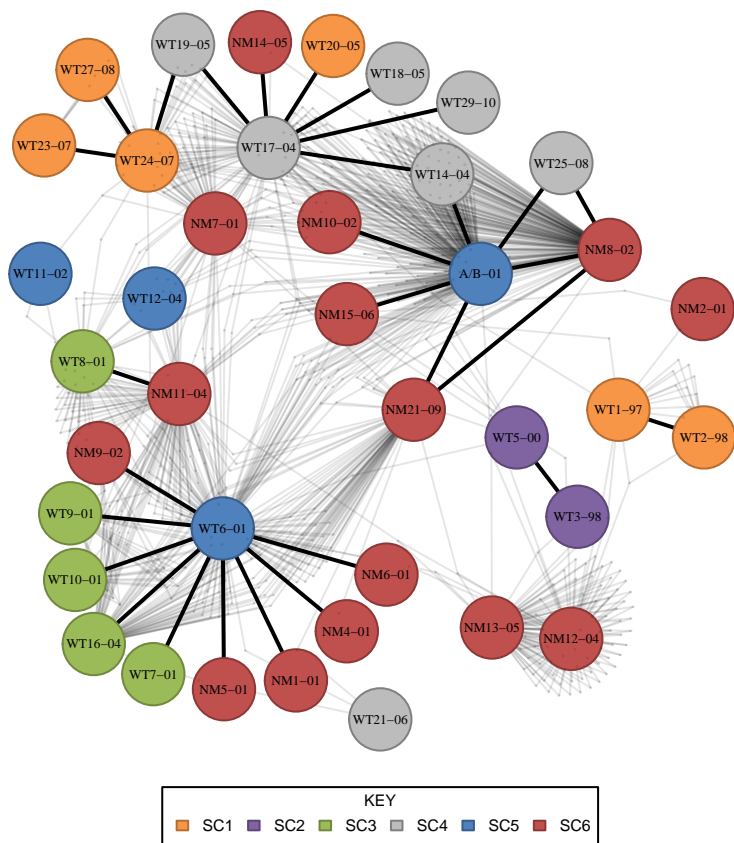

Supplement: Figure S1 — Social network analysis of ON-A subjects. Social network analysis was performed using R statistical software (v3.0.2) with the igraph package. Each large circle represents a single individual colored by their sub-cluster as determined by WGS SNP analysis. Grey lines represent common contacts between study individuals and thick black lines represent direct epidemiological/social-connections between study individuals. (PDF) [file pone.0112928.s001.pdf]
