# Supplementary material for: Marked Microevolution of a Unique Mycobacterium tuberculosis Strain in 17 Years of Ongoing Transmission in a High Risk Population
Source: PLoS One. 2014 Nov 18;9(11):e112928. doi: 10.1371/journal.pone.0112928 (PMC4236100; doi:10.1371/journal.pone.0112928)
Supplement: Material S1 — Supplementary methods and results section. The supplementary methods describe the PCR amplification of regions flanking the 15 kb deletion. The supplementary results describe the WGS results of our laboratory strain H37Rv and how these results were use to evaluate the accuracy of our SNP calling algorithm. Table S1 in Material S1 shows the primers used for PCR amplification of regions flanking the 15 kb deletion. (DOCX) [file pone.0112928.s002.docx]

**MATERIAL S1**

***Supplementary methods, PCR ON-A ^NM^ deletion:*** The ON-A^NM^ variant was defined by a characteristic 15 kb genomic deletion. A custom PCR was designed to evaluate the presence or absence of this region in all ON-A isolates (n=61). Primers were designed to flank the deleted region, so that ON-A^NM^ isolates gave a band of approximately 1kb, while ON-A^WT^ isolates gave no signal (region too large to be amplified). PCR was performed using standard protocols. Primers are described in supplementary Table S1. Following PCR, sequencing of selected amplicons was performed by Sanger methods using standard procedures.

Table S1. Primers used for 15Kb deletion PCR

| Target | Name | Sequence |
| --- | --- | --- |
| 15Kb flanking regions | delF | 5’-GTT CCT GCG TGC TGG -3’ |
|  | delR | 5’-CGC TTG ATC CGG GCG-3’ |
| Rv1366A (MT1413*) | MT1413-F | 5’-ATT CAT GAT CTG CGT GAC CA -3’ |
|  | MT1413-R | 5’-ACC AGT GAA GTA CCG CGA AT -3’ |
| Rv1352  (MT1401*) | MT1401-F | 5’-GAA GGT ATC GGC CAT CAT TG-3’ |
|  | MT1401-R | 5’-CCA GTG GGG AAC TAC CTG AA-3’ |

* In parenthesis is *Mtb* CDC1551 gene nomenclature

**Supplementary results, *Accuracy of SNP calling****:* We identified 91 polymorphisms including SNPs and small indels when compared to the reference genome (NC_000962). Of these 91 polymorphisms, 68 of them have been previously reported in other sequenced H37Rv strains from different labs around the world and had been highlighted as most likely errors in the NC_000962 reference genome.[29] Of the remaining 23 polymorphisms, 6 were shared with 4 H37Rv genomes.[29] This left only 17 novel polymorphisms between our H37Rv strain compared to the published reference genome. However, most of them corresponded to PPE (n=13) and PE-PGRS (n=2) genes which in our analysis workflow are automatically discarded due to ambiguity caused by low coverage on high GC regions.[17,29] In summary, Comparison of our WGS data with available reference H37Rv genomes revealed only 2 curated polymorphisms, a non-synonymous SNP in ponA1 (1888C>T, Pro630Ser), and a transversion in a non-coding region (459399 A>C). Our H37Rv strain is used for routine quality control activities (e.g. sensitive control strain for drug susceptibility testing) and it has been subjected to continuous in-vitro passage which likely accounts for the accumulation of these 2 SNPs. Detection of this small number of changes indicates that our SNP calling workflow is sensitive and robust. Having confirmed the accuracy and quality of our SNP-calling algorithm, we focused on differences observed between ON-A and H37Rv and within the ON-A isolates. One of the 56 sequenced isolates represented a mixed infection with a non-ON-A genotype and therefore was removed from SNP analysis.
